# Exploring Rice Consumption Habits and Determinants of Choice, Aiming for the Development and Promotion of Rice Products with a Low Glycaemic Index

**DOI:** 10.3390/foods13020301

**Published:** 2024-01-17

**Authors:** Diva Cabral, Ana P. Moura, Susana C. Fonseca, Jorge C. Oliveira, Luís M. Cunha

**Affiliations:** 1 GreenUPorto—Sustainable Agrifood Production Research Centre/Inov4Agro, Rua da Agrária 747, 4485-646 Vila do Conde, Portugal; diva.cabral@fc.up.pt (D.C.); apmoura@uab.pt (A.P.M.); susana.fonseca@fc.up.pt (S.C.F.); 2 DGAOT, Faculty of Sciences, University of Porto, 4485-646 Vila do Conde, Portugal; 3 DCeT, Universidade Aberta, 4200-055 Porto, Portugal; 4 School of Engineering and Architecture, University College Cork, College Road, T12 YN60 Cork, Ireland

**Keywords:** rice consumption, consumption drivers, glycaemic index, mixed methods, thematic analysis, rice provisioning process, rice types, healthy rice promotion

## Abstract

Current consumption drivers, particularly those related to health and wellbeing, have been influencing trends for the lower consumption of cereals, particularly rice, due to their typical high glycaemic index (GIs) and consequent impacts on obesity. To satisfy this consumer concern, more food innovations that promote healthy eating habits are required. Such innovations must be consumer-oriented to succeed, understanding the dynamics of consumer habits and responding to consumer expectations. This study explored these habits, from acquisition to consumption practices, and the expectations of the European market from the perspective of the major European consumer, Portugal, to obtain insights that support the development of low glycaemic index (GI) rice products. A mixed-methods approach was applied. For the first quantitative questionnaire, 256 Portuguese rice consumers aged 18–73 years were recruited. Twenty-four individuals were selected according to their gender and rice consumption profiles for in-depth interviews. The results confirmed that rice was the main side dish for the participants and was mainly consumed at home, cooked from raw milled rice. The drivers of consumption differ according to the provisioning process stage. In the acquisition stage, participants reported benefits from the rice’s dynamic market by comparing products on price, brand, and rice types. In the preparation stage, participants reported the adequacy of the recipe and occasion, while in the consumption stage, participants enhanced their sensory preferences, depending on the rice dish. Although the GI concept was unknown to half of the participants, it was perceived as interesting and positive for healthy eating. Consumers showed concern about the taste and naturalness of the product, preferring it to be as close to a homemade dish as possible. The negative perceptions we verified were interpreted to be due to a lack of knowledge about the GI concept. Therefore, awareness actions and informative campaigns are recommended to promote low-GI rice products.

## 1. Introduction

Rice (*Oryza sativa* L.) is a grain widely consumed worldwide. It is the staple food of most Asian and African countries, so its overall world per capita consumption reaches 78.9 kg/year [1]. Portugal has the highest European per capita consumption, with an average of 19.3 kg/year in 2020 compared with an average consumption in Europe of 7.2 kg/year [1]. The fact that Portugal is the fourth largest rice producer in Europe [2] facilitates the availability of this staple food in the market, even though Portugal imports far more than it exports [3].

Cereals were the largest contributor to Portugal’s per capita daily food availability (30.2%), with rice contributing 17.8% of the daily per capita availability of cereals, second only to wheat, a staple food across Europe [4]. Rice accounts for 5.2% of the total energy intake, which is higher than that of its most direct carbohydrate competitors, potato and pasta, contributing 4.6% and 3.0%, respectively [5].

There are two main commercial rice types in Portugal: Carolino, a long-grain Japonica subspecies with kernels over 6 mm in length, a length-to-width ratio of 2–3, and amylose below 22%, and Agulha, a long-grain Indica subspecies with kernels also over 6 mm in length, but with a length-to-width ratio of at least three and amylose above 25%, according to Portuguese legislation DL 157/2017. Other white rice types, such as Arborio and Carnaroli (the Japonica varieties used for Risotto) and the aromatic Basmati and Jasmine, are also becoming increasingly popular [6]. Furthermore, in Portugal, 88% of rice is consumed as white rice, and only 12% is consumed as brown rice [6], like global rice consumption patterns [7,8].

Brown rice results from simply de-husking and sorting the dried paddy rice, thus retaining the bran. It is nutritionally healthier due to the nutritional components primarily contained in the bran, such as lipids, proteins, dietary fibre, γ-oryzanol, and phytochemical components [9]. Consequently, it has been associated with a reduction in the risk of several chronic diseases, including various types of cancer [10,11], the reduction of blood cholesterol levels [12,13], and the ability to decrease the risk of type II diabetes [14,15]. However, brown rice is less accepted than milled rice (which results from the removal of the bran and germ by abrasion and subsequent polishing, and is also known as white rice) in terms of texture (brown rice is harder and chewier than white rice) [16] and flavour (due to lipid oxidation that leads to the development of off-flavours) [17]. The milling process improves these sensory properties by removing the bran and germ, leaving only the endosperm, which is mostly carbohydrates (CHO). As the most nutritious parts of the grain are removed, milled rice is left with very few essential nutrients other than starch, but it is better accepted by consumers due to its colour, texture, and flavour, especially for aromatic rice varieties, such as Basmati and Jasmine [16,17,18]. Furthermore, it is considered more convenient to use as it cooks in less than half the time required to cook brown rice. However, due to the amount and nature of its refined CHO, milled rice has a high glycaemic index (GI) [19,20].

The GI indicates the health quality of the CHO present in foods, based on how quickly blood glucose levels rise following digestion. Based on the GI, considering glucose as a standard food, foods are considered as presenting low GIs (GI ≤ 55), intermediate GIs (56–69), or high GIs (GI ≥ 70) [21,22]. Foods with high GIs potentiate a rapid rise in blood glucose; low-GI foods tend to release glucose more slowly and steadily. Thus, low-GI foods are considered healthier, improving blood glucose and lipid control, and promoting insulin sensitivity, and are therefore beneficial dietary treatments for diabetic patients [23,24,25].

Based on the international GI table of Atkinson et al. [21], the GI of cooked rice ranged from 19 to 116. In this compilation, no entry of Portuguese rice was found; however, there were indications of several milled rice of the japonica subspecies, which presented values between 76 and 89. This subspecies, the most locally produced rice in Europe and Portugal, had the highest GI. Portuguese japonica varieties *Ronaldo* and *Ariete* have GI values of 89 and 151, respectively [26]. GI depends on various factors such as variety, processing, and recipe ingredients [27,28,29,30]. 

According to the International Diabetes Federation, an estimated 463 million people globally live with diabetes, and this number is projected to escalate to 700 million by the year 2045 [31]. Portugal is the second country in the European Union with the highest prevalence of diabetes (13.6% of the population aged between 20 and 79) [32]. Several nutritional studies have shown that the excessive consumption of white rice is associated with an increased risk of non-communicable diseases, namely diabetes, hypertension, obesity, and cardiovascular diseases [33,34,35,36,37,38,39]. Many studies have discussed GI and its importance in controlling diabetes and related diseases and demonstrated the importance of a food-centred approach [29,40,41,42,43,44,45]. Methods to reduce the GI of rice have been investigated, and products with a lower GI have been obtained by controlling the processing parameters [46,47,48,49,50] using selected rice varieties and adding ingredients [51,52,53,54]. These trends have pressured the food industry to develop low-GI rice products in Western societies, where health has functioned as an important criterion for individual food choices [55]. Given the scientific evidence of the usefulness of GI in controlling the metabolic syndrome and the different methods of obtaining products with a lower GI, it is important to investigate this topic from a consumer-centred perspective. This will support a better promotion of healthier habits of rice consumption, particularly in countries with high rice consumption. Such steps involve the understanding of consumers’ perceptions, behaviours, and attitudes, as well as the development of rice-based food products that meet the consumers’ expectations.

This study aimed to explore and understand the rice-provisioning process of Portuguese consumers, from acquisition to consumption practices, and their associations and consumption drivers regarding low-GI products. The approach was to gather insights to support the development of new consumer-oriented low-GI rice products and healthier forms of rice consumption while focusing on the European market with the highest per-capita consumption.

## 2. Materials and Methods

A mixed methodology [56] was applied, combining the evaluation of consumers’ behaviour, assessed through self-reported measures questionnaires, and in-depth interviews. The combined use of these procedures in consumer research allows for an apprehension of the phenomenon and the object of study from different perspectives [56], thus inter-validating the findings. All participants followed an informed consent procedure before participating, previously approved by the Ethical Committee of the Faculty of Sciences of the University of Porto, with reference number 50/2023. All participants received a small financial compensation for their participation.

### 2.1. Questionnaire

A structured self-reported electronic questionnaire was applied using the Lime Survey software v.2.50. The questionnaire (Supplementary Materials) combined indirect and direct methods, respectively: (i) free word association (FWA) towards low-GI foods, (ii) rice preparation and consumption practices, and (iii) sociodemographic characteristics. In the FWA task, participants were invited to write down the first three words that came to mind when thinking about “low glycaemic index foods” (*alimentos com baixo índice glicémico*, in Portuguese). To avoid biases from previous questions, the FWA task was the first to appear in the questionnaire flow. In the second part of the questionnaire, the frequency of rice consumption, the place of consumption, the rice dishes consumed, rice substitutes, and rice purchase formats were evaluated through a checklist-type multiple-choice questionnaire. Seven response options were considered (in general and by rice type) for consumption frequency evaluation: (i) less than once a month; (ii) 1 to 3 times per month; (iii) once a week; (iv) 2 to 4 times a week; (v) 5 to 6 times a week; (vi) daily; and (vii) 2 or more times per day. The types of rice present in the Portuguese market were included in the questionnaire options under their trade names: *Agulha* (long grain Indica variety), Basmati, brown rice, *Carolino* (long grain Japonica variety), Jasmine, parboiled, and Risotto (although Risotto is not a type of rice but a form of cooking, it is known by consumers as “Risotto rice” short for “rice for Risotto”, with very few consumers being able to relate to the varieties widely used for Risotto cooking (Arborio or Carnaroli). Both dishes served as a side (accompanying some protein source on the side) or as a main course (mixed with the protein source) were considered in the evaluation of the frequency of consumption, such as plain rice, rice with duck, rice with seafood, rice with carrot, rice with tomato, rice with fish, rice with beans, rice with peas, rice with chicken, rice with cabbage, rice with meat, and rice with chicken blood. The latter, designated as *Cabidela*, is a traditional dish of the gastronomy of the northern region of Portugal, made with *Carolino* rice, chicken and its blood, and vinegar, where the meat and offal are also incorporated, resulting in a stew served as a main course [57]. These rice dishes were chosen due to their popularity in Portuguese cuisine, based on a multitude of blogs, cooking magazines, and videos of chefs and home cooks, among other varied sources of popular knowledge related to traditional Portuguese cuisine, and providing a much wider variety of rice dishes than what exists anywhere else in Europe. Some dishes involve a very loose grain and dry dish (e.g., a typical Basmati), others a very creamy one (e.g., a typical Risotto). The questionnaire was finalised, asking for age, gender, educational level, household size, and monthly household income (all other details remained anonymous). The questionnaire was completed in person using a computer at the premises of the recruiting company. Data were collected over a two-month period, from February to March 2018.

#### 2.1.1. Participants 

Participants were recruited from the sensory evaluation company Sense Test’s consumer database (Vila Nova de Gaia, Portugal). They were mainly residents of the Porto metropolitan area, in the North of Portugal, which has the country’s highest per capita rice consumption. 

A convenience sample of over 250 consumers was recruited from the database, with the following inclusion criteria: (i) being Portuguese, (ii) consuming rice at least once a month, and (iii) being responsible or sharing responsibility for grocery shopping and preparing meals.

#### 2.1.2. Data Analysis

In the FWA task, response words were classified into categories, which were then classified into dimensions, considering internal homogeneity and external heterogeneity, using the triangulation technique [58]; it followed the same analytical procedure described in a study by Cabral et al. [57]. After the individual categorisation of the response words done by experienced researchers, the agreement between categorisations was verified and a label was assigned to each category and dimension. The frequencies of each category/dimension were determined by counting the participants who mentioned that category/dimension, without considering whether the same respondent evoked the words of the same category/dimension or not; so, the sum of the frequencies may have been greater than 100%. The cut-off point of 5% of participants, by category, was considered as inclusion criteria for further analysis. To assess the relationship between the participants’ characteristics and the type of perception in relation to low-GI foods, an independence χ^2^ test was performed. When significant differences were found (significant level of 0.05), a χ^2^ test per cell was used to identify the source of the global χ^2^ variation [59]. The words/categories/dimensions gathered from the categorisation of the FWA task were translated into English using a back-translation procedure [60].

To assess the frequency of the consumption and purchasing of rice, the response options were converted to obtain the weekly frequency using the following conversion factors: 0 (less than once a month), 0.5 (1 to 3 times per month), 1 (once a week), 3 (2 to 4 times a week), 5.5 (5 to 6 times a week), 7 (once a day) and 14 (2 or more times per day). Descriptive statistical analysis was performed to determine self-reported consumption frequencies, means, standard deviations (SDs) and standard errors (SEs). Statistical data analysis was processed with XL-STAT^®^, v. 2020.5.1 (Addinsoft, New York, NY, USA).

### 2.2. Interviews

A face-to-face individual interview was conducted to provide a more detailed understanding of consumers’ experiences and opinions about rice choice, preparation, and consumption practices, and a better understanding of the associations and consumption drivers regarding low-GI products [61]. For this, a semi-structured interview guide of open-ended questions (Supplementary Material) was developed considering the following dimensions: (i) rice choice criteria and consumption habits; (ii) rice sensory criteria valorisation; (iii) knowledge about foods with low GI and drivers of choice of rice with low GI; and (iv) expectations regarding new rice products, to promote low-GI alternatives. After probing participants about the GI concept, they were presented with the FAO/WHO definition [62], in the following simpler version: “The glycaemic index is defined as the increase in blood glucose after eating a certain food”, and some examples were given to the participants. Examples of high-GI foods include white wheat bread, potatoes, and sugary desserts [21]; while low-GI foods include fresh fruits and vegetables, such as apples, pears, and beans [21]. The first two authors conducted the interviews during September 2019.

#### 2.2.1. Participants

Twenty-four participants who responded to the initial questionnaire were chosen for a semi-structure face-to-face interview based on their willingness to participate and on their reported rice consumption profiles: (i) daily rice consumers (eight participants who consume rice at least once a day), (ii) light rice consumers (eight participants who consume rice less than three meals per week), and (iii) brown rice consumers (eight participants who consume at least three brown rice meals per week). According to Cabral et al. [61], the rice consumption profile influences perceptions regarding “rice” and “rice with low GI”. An attempt was made to have male and female participants in each of the three groups, participants with and without higher education, and participants from different age groups, to minimise the eventual impact of major variations in such demographics. The interviews were conducted in a separate room with a see-through mirror window for observations at the consumer studies company Sense Test, Lda. All interviews began with a greeting and initial introduction as an icebreaker, followed by a report on their two meals (lunch and dinner) during the past week. The interviews were audio and videotaped with the stated consent of the participants. The interviews had an average duration of 32 ± 10 min (maximum duration: 61 min; minimum duration: 17 min).

#### 2.2.2. Data Analysis 

The transcripts of the interviews were explored with a qualitative data analysis software, QSR NVivo2020 (Copyright^®^ QSR International Pty Ltd., Melbourne, Australia). Interviews were analysed using a thematic analysis procedure that involves a progression from description to interpretation data [63]. A comprehensive data-coding process and identification of dimensions, consistencies, and discrepancies across dimensions, was applied to provide an in-depth understanding of the texts [64]. To illustrate the analysis, consumer direct quotes were transcribed, describing the topic explored.

## 3. Results

### 3.1. Questionnaire

#### 3.1.1. Participants’ Characterisation

Appendix A (Table A1) shows that the 256 participants responded to the full questionnaire. The participants were mainly female (64%), with a mean age of 40 ± 12.8 years old (ranging between 18 and 73 years old), spread across three age groups, with 64% of participants being without a higher educational level. 

#### 3.1.2. Questionnaire on Rice Preparation and Consumption Practices

Table 1, Table 2 and Table 3 provide information about participants’ self-reports on their preparation and consumption of rice. Participants regularly consume rice every week with an average rice consumption of 4.4 times/week. Most of the participants (53.9%) consumed rice one to four times/week, 39.8% consumed five or more times/week, and only 1.2% consumed less than one time/week (Table 1).

The most-consumed type of rice by the panel was *Agulha* (long grain Indica, 2.6 times/week), followed by *Carolino* (long grain Japonica, 1.9 times/week) (Table 1). Basmati rice was consumed on average 1.1 times/week, closer to parboiled rice (0.8 times/week). Participants self-reported that they consume less brown rice (0.5 times/week), Jasmine rice (0.4 times/week), and Risotto rice (0.3 times/week). 

On average, rice was mostly consumed at home (4.1 times/week) rather than out of the home: 1.0 times/week at restaurants and 0.8 times/week at canteens/refectories (Table 2). Approximately 65% of the participants consumed rice at home one to four times/week, and 34% consumed rice at home five or more times/week. Of the 186 subjects who answered that they chose rice in restaurants, 57% reported doing so less than one time/week. Regarding canteens, only 52 respondents chose rice when they ate in them, and of these, the majority (61%) did so one to four times per week. 

**Figure 1:**
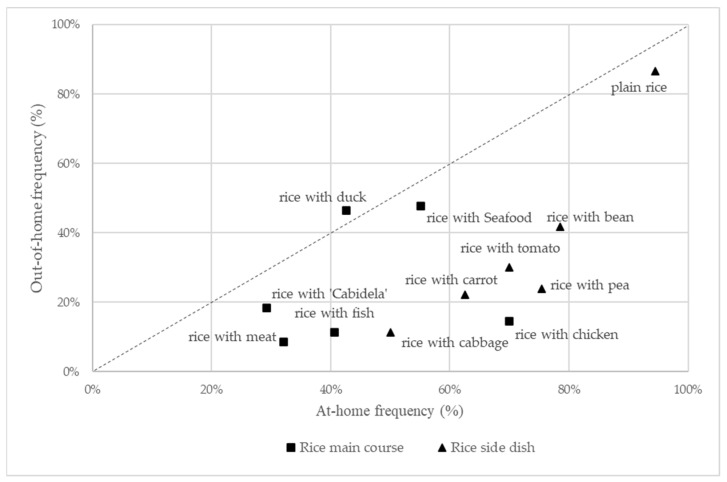
Frequency of participants (%) that self-reported rice consumption at home and out of the home, according to rice dishes. Sample size = 256.

Plain white rice was reported as the most frequently consumed rice dish, both at home and out of home (see Figure 1). All rice dishes being analysed were consumed more frequently at home than out of home, except for rice with duck.

Table 3 shows the purchase frequency of the various rice commercial formats consumed at home by the panel. Raw rice was bought by 42.3% of respondents less than one time/week to cook at home, and 77.3% of respondents bought ready-to-eat rice at the same frequency. Most participants indicated that they had never bought rice in dehydrated (86.3%), pre-cooked (82.8%), frozen (90.6%), or refrigerated (88.7%) formats, demonstrating the low popularity of ready-to-eat or processed rice among participants.

#### 3.1.3. Questionnaire on Free Word Association with “Low Glycaemic Index Foods” Stimulus

**Figure 2:**
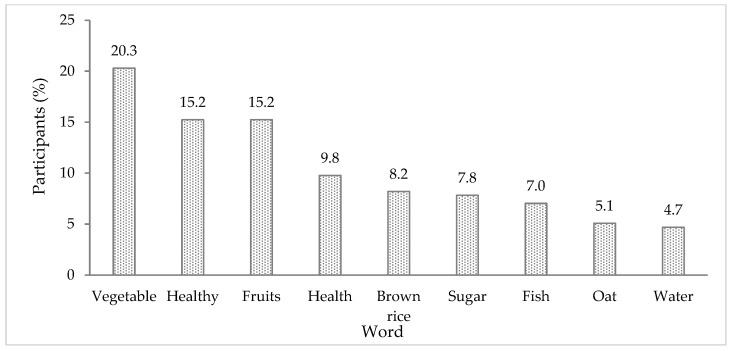
Frequency of participants (%) citing the ten most evoked words for the stimulus “Low glycaemic index foods”. Sample size = 256.

The free word association generated 719 words, of which 260 were different response words, with 49 participants (17.2% of the total) not responding. Figure 2 presents the ten words most frequently associated with “Low GI foods”. Among the most frequently mentioned words were foods that indeed generally have a low GI, such as “vegetables”, “fruits”, “brown rice”, “fish”, “oat”, and “water”. “Healthy” and “health” were also the main perceptions concerning this stimulus.

The words were grouped into nineteen categories, four of which did not meet the minimum answer criteria—5% of participants—to be included in the analysis: sweetener, price, negative emotions, and family. These were then merged into eleven dimensions (Table 4). The dimensions that were most mentioned were “Fruits and vegetables” (40.2% of participants), “Nutrition” (38.7% of participants), “Health” (32% of participants), and “Whole grains” (21.5% of participants).

In the “Sugar reduction” and “Nutritional aspect” categories (“Nutrition” dimension), participants defined their understanding of low-GI food or a low-GI diet. Expressions such as “eat little sugar”, “little sugar”, “low consumption of sweets”, “no sugar”, “no glucose”, and “low carbohydrates” were referred to. Although these categories have reflected some knowledge about the low-GI concept, there were some incorrect associations, which were grouped into the category “Low GI disconnects”, where 8.2% of participants associated ideas related to fat and salt reduction, the addition of vitamins and iron, into their understanding of a low-GI food.

The “Health” dimension clearly reflects the idea of low-GI foods, which has been assigned a function of control and maintenance of good health. In its “Physiological” category, terms associated with biological functions related to sugar ingestion, digestion, and the effect of blood glucose on the body were gathered. In the “Positive attitudes and emotions” dimension, ideas of acceptance and positive emotional state were expressed beyond the terms that generally qualify the stimulus in a positive way, such as the words “necessary”, “ideal”, “positive”, and “decisive”. However, in the “Sensory” dimension, the associations were not very positive in relation to flavour, associating words such as “tasteless”, “bitter” and “sour”, which are attributes that are not normally appreciated by consumers.

The categories resulting from associations with the stimulus “Low GI foods” were significantly dependent on gender (χ^2^ = 22.157, DF = 11, *p* < 0.05), age group (χ^2^ = 37.802, DF = 22, *p* < 0.05), and educational level (χ^2^ = 16.205, DF = 11, *p* < 0.05). Table 5 shows the result of the χ^2^ test per cell. Women associated “Fruits and vegetables” with “Low GI foods” significantly more than men.

Older participants expressed more “Positive attitudes and emotions” than younger participants, which in turn mentioned significantly more “Sensory” and “Nutritional aspects”. In the “Sensory” dimension, the descriptors elicited were negative, which shows that while young people have a less positive perception of “Low GI foods”, the older ones have the opposite perception, attributing significantly more words referring to “Positive attitudes and emotions”, showing more awareness of the health benefits that low-GI foods can bring to the consumer.

As for the level of education, participants with higher education made significantly more associations with “Whole grains”, while those with no higher education made significantly more associations with “Bread and pasta” and “Low GI disconnects”. 

### 3.2. Interviews

The interviews evaluated two main broad dimensions: the domestic rice provisioning process and the perception towards GI concepts and low-GI foods. Six major levels of analysis were identified: (i) acquisition (the rice choice criteria), (ii) cooking (including aspects of preliminary preparation and cooking), (iii) eating, (iv) GI concept, (v) the drivers and barriers of choice for low-GI rice, and (vi) new low-GI rice alternatives. This approach follows the sequence of stages in which the consumer goes through domestic rice provisioning, from the initial rice purchase decision to the eating stage, rather than simply focusing on eating in the narrowest sense of consumption [65].

#### 3.2.1. Participants’ Characterisation

A total of twenty-four consumers, eight from each of the rice consumption profiles, as previously described, were recruited for the interview task, as shown in Appendix A (Table A2). Once relevant information regarding the phenomenon under investigation was obtained, and the data saturation was achieved, the sample size was deemed adequate [66].

#### 3.2.2. Acquisition

Various factors influenced the choice of raw rice, ranging from practical reasons (price, brand, rice variety) to physical appearance and personal concerns (health, nutrition).

The participants benefit from rice’s dynamic market by comparing products on price, brand preferences, and packaging, and according to the rice varieties. Consumers can only choose foods which are available and that they can afford to pay for. Participants also mentioned that they changed their traditional variety of rice choices by replacing the traditional Carolino with *Agulha* and other exotic rice, such as Basmati and Jasmine, due to their appealing taste, texture, appearance, and speed of cooking: ‘… *much more Agulha rice and Basmati* (…) *I always choose Agulha or Basmati because I don’t like Carolino very much*’, P1; ‘… *more recently it is Basmati rice, but before that, it was Agulha rice*’, P16. However, those who frequently consumed brown rice (Group 3) complained that there are not as many brands for this type of rice and that the product is not well displayed on supermarket shelves, hidden among other dietetic products: ‘… *within the brown rice I don’t find great varieties of brands*’, P21. This suggests that brown rice would be purchased more often if brown rice were in the same place as milled rice. Furthermore, the price was reported by most participants as a decisive element in rice purchase (‘*Price is always first*’, P10), and some of them tried to join the desired attributes with the affordable price (‘*The quality and the price, I try to reconcile these two things*’, P18), by benefitting from sales promotions, particularly price reductions. They reported buying the manufacturer’s brands on promotion or trying new types of rice (‘*It was only when promotional campaigns began to appear that I started to consume and try new types of rice. I also didn’t know about parboiled until one day I saw it on promotion, and it caught my attention*’, P2; ‘*If the parboiled from X* [manufacturer’s brand] *is not on promotion, I won’t buy it. I can even buy the Agulha*’, P9) or increase rice quantity (‘*When I get a promotion, I buy the X* [manufacturer’s brand]. *I buy about three or four kg to have at home*’, P14; ‘*If it’s on the promotion of the brand I like, I bring more quantity*’, P5).

The promotional campaigns stimulated purchases not only due to lower prices but also because the products are strategically displayed in the store to increase consumer interest: ‘*There is always something yellow there [commonly used promotional tags] that catches our attention* (…) *in the middle of all the shelves it is often very difficult to find the rice we are looking for. We often go for the packaging, for the colour of the packaging, but if there’s a promotion, it’s yellow or whatever and that one stands out*’, P21. This allows consumers to become less loyal and more price sensitive, switching from brand to brand and type of rice depending on the benefits offered: ‘*I use several brands and qualities of rice*’, P5; ‘…*if there is a big difference in price, I buy the cheapest one* [manufacturer brand] *or the own-label one*… *and if* [own label] *is cheaper than the cheapest brand, I always buy it*’, P16.

Participants also reported that when they buy rice, they particularly focus on the visual appearance of the grains, which can be evaluated in terms of the colour, size, and shape of the grains, as mentioned by this participant: ‘… *the colour is very important and* [the grain] *shouldn’t be too small, it must be an acceptable size rice*’, P5. Depending on the rice variety, when making their purchases, they pay attention to a particular sensory attribute compared to others: ‘… *for Basmati rice, for example, I look at the grain. The length and breadth of the grain will tell me if that rice is worth taking. Carolino rice, the grain is shorter and more rounded* (…) *I waste a lot of time on choices*’, P5. 

Nevertheless, some participants (*n* = 6) consider that carbohydrates in general, and rice in particular, negatively affect health, namely, as a cause of weight gain, as reported by this participant: ‘*Calories, carbohydrates, that’s what I mean* (…) *I avoid carbohydrates, rice, and potatoes* (…) *She* [daughter] *is entering the stage of looking in the mirror* (…) *we have been avoiding rice* (…) *I avoid eating those carbohydrates at night, I try to put on more soup to satisfy us* (…) *if we eat a lot of rice* (…) *then losing weight is very difficult*’, P9. By contrast, brown rice, parboiled, and wild rice were positively related to a healthy diet, and most of these associations were made by the “brown rice consumer” group: ‘*We always try to opt for brown rice or parboiled rice, which is rice that has more nutrition than milled or polished rice. We rarely buy milled rice* (…) *I see that it’s brown rice and I take it, sometimes I don’t even look at the price*’, P23. In the same way, brown rice is perceived to be natural, contrasting with polished rice, which is no longer natural.

Some participants emphasised the importance of the origin of rice, indicating their preference for national rice (‘… *and I pay attention beyond the price if it is Portuguese rice* (…) *I try to give priority to what is Portuguese*’, P5), referring to some geographic areas of national rice production (‘… *I buy national rice* (…) *I try to choose the rice produced in Portugal. Mondego, Sorraia, I don’t know, Alentejo, river Sado*…’, P11). These participants had a very positive evaluation of national rice, associating it with better quality (‘… *go to another store* (…) *to buy a better one, and I think it’s Portuguese*’, P18), and a connection to naturalness and more artisanal and sustainable agriculture: ‘… *I don’t throw it* [rice] *away because I know how much work it takes to harvest each grain, especially here, which is a very manual task*…’, P5; ‘… *I look for rice produced in Portugal* (…) *that grows slowly, without large amounts of chemical products. I prefer it because it has fewer chemicals*’, P11. 

#### 3.2.3. Cooking

Rice plays a relevant role in Portuguese cuisine, as the participants considered it an everyday food (a ‘staple’ food) and an ingredient served as a side dish or a main course. Different varieties are available on the Portuguese market, both of local production and those imported. This assortment allows wide culinary applicability: ‘*Sometimes Carolino, it depends, depends on the dishes. Agulha rice I even use more if it is, for example, rice with duck, rice in the oven*’, P12; ‘*Everyone likes rice. I use any rice. Carolino, Agulha, Basmati, Parboiled, Risotto, Brown rice* (…) *I diversify a lot*’, P17. Participants also reported that the consumption of rice adapts to the daily consumption situation, depending on i) the season of the year (‘… *in winter I eat a lot more rice than in summer but that’s just because I like. Because in winter it tastes much better, it’s warmer. In summer I go more for salads and vegetables*’, P21); ii) its use for a special moment, such as Sunday family lunch day or festive season day; or iii) for everyday dishes: ‘… *during the week I make brown rice* (…) *on Sunday I make the normal one* [milled rice] *for everyone*’, P9; ‘*I’ve been making Basmati when it’s something like that better* [special occasion]’, P10. 

The convenience was particularly associated with *Agulha*, Basmati, and parboiled rice, contrasting with brown rice and *Carolino*, where a certain lack of culinary skills was shown by our participants, reporting that this makes it difficult to reach a good rice dish: ‘*I think* [*Agulha*] *it’s much easier to cook and it also comes out looser* (…) *I have to leave lunch for my husband; it’s much easier to reheat rice like this* [*Agulha*] *than a Carolino*’, P1. In the case of brown rice, in addition to the lack of cooking skills, some participants also mentioned the long cooking time as another barrier to using this type of rice in their daily life: ‘*It’s very difficult* [to cook brown rice], (…) *I’ve tried it in several ways, and it is not the same* [like the restaurant where I usually eat]’, P20; ‘… *brown rice* [I buy] *too, but less.* [because] *It takes longer to cook*’, P10. 

Participants also reported that they used simple tricks when cooking rice to get the desired dish, as a safety measure, or to simplify the cooking process, namely: i) removing rice impurities; ii) soaking rice grains before cooking, so that they can release any toxic or hard-to-digest substances as well as shortening the cooking time and cooking uniformly; or iii) regulating the hot temperature to obtain loose rice (‘*If it’s on an electric hot plate when it raises the boil, I immediately turn off the rice, because I don’t like sticky rice*’, P17).

Participants mentioned adding other ingredients to rice dishes, such as vegetables, pulses, and chorizo: ‘Very rarely I’m able to make simple rice, either we put carrots, or we put peas or a little bit of chorizo. It is usually always with vegetables’, P19. Malandro rice is a typical gastronomic practice of Portuguese cuisine, traditionally made with Carolino rice (long grain Japonica variety) cooked in plenty of water, which results in a creamy dish with a lot of broth rich in flavour derived from the added ingredients, such as vegetables, meat, fish, or seafood [57]. It is mostly referred to as Malandrinho by participants due to the influence of the brand name of a popularly known brand of Carolino rice. For some of the participants, the intention to add other ingredients is to improve the nutritional content of the dish: ‘I try to put in more vegetables than rice to make sure we eat as many vegetables as we need’, P5; ‘I usually add carrots or peas or beans, and it becomes more nutritious’, P6.

In the case of brown rice, the intention is also to mask or improve its flavour. ‘…brown rice doesn’t have the same flavour as Agulha rice, for example, if it’s plain, my son won’t eat it (…) as it has a characteristic flavour, it can be masked [with the addition of other ingredients]’, P1. Nevertheless, participants considered this practice ineffective, as this type of grain has some difficulty absorbing flavours from other ingredients: ‘… it gets a little rawer and does not seem to absorb as much water and everything else. Of course, it will have less flavour…’, P16. 

#### 3.2.4. Eating

Participants reported that they like rice so much that some of them or their family members eat rice daily: ‘*They all like and eat rice! If they can, they eat rice every day*’, P14. Others reinforce this preference by putting rice as their favourite side dish, compared to other alternatives: ‘… *I prefer rice to French fries* (…) *it’s my favourite side dish. I’ve always loved rice*’, P4. Others also mentioned that they choose the type of rice according to their taste preferences: ‘*I really like Basmati rice, it’s a rice that tastes good to me*’, P4. In this context, the cooked rice grains’ appearance, flavour, and texture were the main sensory attributes identified by participants regarding their dish preferences. Independently of the dish, participants expressed their preference for whole kernels and grains with good extensibility (swelling of the cooked grain): ‘… *The growth* [swelling] *of the grains* (…) *at the end of the cooking process has a nice appearance, and it becomes thick. It’s how I like rice*’, P1.

For brown rice, different sensory descriptors emerged, such as hard, dry, dark, brown, dirty, and tasteless, explaining their dislike due to these sensory characteristics: ‘… *brown rice is a little darker, I like it less*’, P4; ‘*I don’t like brown rice, it’s tasteless, girls* [wife and daughter] *like it anyway*’, P13; ‘*It looks like it gets a little rawer* (…) *I think that’s why* [I do not like brown rice]. *Because it doesn’t have as much flavour, and the texture too* [I do not like it], *I think it’s more* [because of] *the texture*’, P16. It should be noted that none of these participants belonged to the brown rice consumers group, although these also recognise the fewer appealing attributes. However, they feel motivated to consume brown rice because of its health benefits: ‘*It’s not because of the flavour* [that I eat brown rice], *it’s because of the information I have that it would be healthier. It wouldn’t be for the taste because it’s not very pleasant*’, P24.

#### 3.2.5. Glycaemic Index Concept

When the concept of the glycaemic index was introduced, half of the participants declared not knowing about the concept. Several made erroneous associations with nutrition, thinking that the GI was synonymous with the food “calories”: ‘*It will be the so-called calories that we attribute to food* (…) *general values of, namely, fats, saturated fats*…’, P6, or that it was related to fat content: ‘… *it has to do with fat. It is the fat index. In this case, it will be the fat index of the rice*’, P24. 

To explain the low-GI concept, some interviewees associated it with familiar terms and ideas such as sugar and carbohydrates: ‘*It has to do with sugars* (…) *to have few hydrates. Not having high levels of carbohydrates*’, P1; and gave simple explanations like: ‘*It is the sugar index that we have in the blood*’, P5; ‘*It’s what raises our blood sugar* (…) *whoever has diabetes has to be careful with that, right?*’, P10. Only fewer (*n* = 3 of 24) gave a slightly more complex explanation relating the GI to carbohydrates and digestion: ‘… *foods with a lower glycaemic index release sugar more slowly, and a food with a high glycaemic index even makes us want to eat faster*’, P20; ‘*It’s basically how far food takes our blood sugar levels. The higher the glycaemic index, the worse the food, because it doesn’t leave us feeling satiated for so long, so it’s better than the glycaemic index is not so high so that satiety is longer*’, P21. They were from the “brown rice consumer” group who, as mentioned above, considered health concerns when choosing rice. 

When asked specifically about the GI of rice, most (*n* = 15 of 24) responded that they have never thought about GI in rice, focusing their concerns for this concept only on sweet foods: ‘*I don’t think about it. We will think of other foods like marmalades, and jams. Not for rice*’, P10. In this debate, some participants (*n* = 5 of 24) considered the impact of rice processing on GI, mentioning that brown rice would have a lower GI than milled rice: ‘… *it depends on the rice, doesn’t it? I am aware that the glycaemic index of milled rice is much higher than that of brown rice*’, P21; ‘*A rice with fibre will give us energy for longer. Basically, it will keep us fuller for longer. Refined rice has no fibre, so the glycaemic index is higher*’, P23. Among these participants, some even mentioned possibilities in the search for rice with a lower GI: ‘… *I look for one with a low glycaemic index. Brown rice, for example, has more fibre and so on, a low glycaemic index, but as it takes a little longer to cook when we don’t have that time, we use parboiled rice or Basmati rice*’, P23. Participants who were more familiar with the GI concept applied to the different rice types had higher education or belonged to the “brown rice consumer” group, revealing knowledge and, therefore, health concerns.

#### 3.2.6. Drivers and Barriers of Low-GI Rice Choice

When asking participants about their opinions regarding what would make them buy rice with low-GI instead of other rice types, not surprisingly, health concerns emerged among the 22 participants that discussed this subject: ‘*I would buy* [low-GI rice] for health *reasons, and not to get fat’*, P9. Nevertheless, only a few participants (*n* = 2 of 22) stated that they would choose a low-GI product only in case of medical recommendation because they think that, for instance, a rice product with a low GI would be too expensive and that the investment would only be justified if it is something essential for health: ‘… *if I go to an appointment and the doctor tells me I must eat. I would eat*’, P7; ‘… *if I perceive that it is more beneficial, and the price difference isn’t much*’, P16. 

Participants also demanded convenient solutions for rice with low-GI products, particularly ready-to-eat products (‘*for me a quick meal because of time saving and functional*’, P3), and ease of handling (‘*May it be easier to take with you* (…) *to carry in your bag daily*’, P6). Nevertheless, others were suspicious regarding these potential convenient solutions because they consider that convenience makes products more expensive, or because they believe that the speed of preparation can compromise the typical flavour of rice (‘… *being ready-to-eat, the possibility of being able to be made in the microwave also helps us, oh and that it doesn’t lose the flavour because it is made quickly*’, P16). As the participants idealised these products as convenient and healthy, they perceived them to be expensive because increasing convenience (time savings, ease of preparation, energy savings, etc…) adds value to the final product.

Participants declared their interest in the product with a low GI but stressed the importance of maintaining the genuineness of usual rice dishes: ‘*Just because it has a low glycaemic index is already a factor of interest. But, after trying it, it may be interesting if it resembles other [conventional] products or if it tastes good*’, P13. Some participants also reported that their interest in this type of rice would be a function of its sensory attributes (*n* = 9 of 22) and price (*n* = 7 of 22). Participants may accept low-GI rice products if they are deemed tasty, have a good appearance, and if they are sold at affordable prices. 

Despite participants conceiving of rice with a low GI as a convenient product, some also mentioned naturalness as one of the drivers of choice (‘*I look for the most natural product possible*….’, P23; ‘*Everything that is refined is very artificial. Rice, when it leaves the field, is not white*’, P20; ‘… *being processed loses quality, so if I could eat rice every day, I would eat this* [brown rice]’, P11. Moreover, this concern is related to both the nature of the raw material (‘*A Bio Snack, with Bio products*…’, P18) and the processing technology used: ‘*I avoid things that are processed* (…) *it is like this, the more preservatives things have, the more this and that, it is to be avoided* (…) *that does not have many additives*’, P10. 

Some participants claimed that additional information about this new rice concept should be on the packaging and in advertisements: ‘… *what you were buying, you would be able to buy*’, P3. Within this regard, some participants mentioned that communicational campaigns should also consider practical cues regarding how to cook rice with low-GI meals, namely by including recipes: ‘*Maybe* [I do not buy] *because I don’t know how I’m going to use it*’, P9; ‘… *but can I use it like this? You know, those questions we ask ourselves in the supermarket* (…) *If you have this information in the back like the recipes, it helps*…’, P15. In addition, they consider it important to clearly state the composition and benefits of the product: ‘*If I’m satisfied with the nutritional table, ok. If not, I’ll go to see what’s in the ingredients, and if there’s a name I don’t know, I’m a little bit wary of* (…), *in this case, I always pay attention to the type of initial rice* [base ingredient]’, P21. 

#### 3.2.7. New Rice Products 

As for rice-based products, most participants indicated that they have already consumed some rice products that exist on the market, such as puffed rice cake, crackers, vegetable drinks, and rice-based ready-to-eat meals, and suggested products that they would be interested in purchasing, so long as they do not compromise the food’s sensory appeal. Some examples of these new rice products mentioned were rice desserts and dairy substitutes, convenient ready meals of rice/rice side dishes, or healthy rice snacks (Table A3). Participants expect these new rice products/meals to be convenient, healthy, and close to conventional products/meals. The dairy products listed on the FWA could be suggestions for low-GI foods that participants wanted.

## 4. Study Limitations

Despite the questionnaire sample size and the effort to recruit participants with a meaningful rice consumption pattern, the sampling approach did not follow a probabilistic design, meaning that care should be taken if extrapolating for the whole Portuguese population. 

## 5. Discussion

### 5.1. Rice Consumption Habits

The survey results emphasise that rice continues to be an important part of Portuguese cuisine. In contrast to most European countries, rice is an almost everyday food for Portuguese consumers. The average rice consumption for French and Spanish consumers is only 0.9 times/week [67]. For the British, rice is considered “neither an everyday food nor a special food” [68], while for the French, it is considered “an exotic food” [69]. Regarding the type of rice, *Agulha* is the most consumed, followed by *Carolino* and Basmati. A previous study suggested that individuals with the highest rice consumption regularly chose *Agulha* rice, whereas those with the lowest weekly intake preferred Basmati and *Carolino* rice [57]. The participants’ narratives were consistent with those results, indicating that *Carolino* rice has been partially replaced by *Agulha* rice and Basmati rice has replaced *Agulha* rice.

Parboiled, Jasmine, and Risotto were consumed less than one time per week. This consumption pattern tends to mimic the actual national pattern of usage. The most comprehensive collection of sales data is done by the Nielsen company, which for the 12-month average, from March 2023, of rice consumption in Portugal gives: 47% *Agulha* rice, 22% *Carolino*, 13% Basmati, 11% parboiled, 1.3% jasmine, 0.9% risotto, 0.7% brown, and 4% other types (percentages of tonnage sold in the country, Nielsen, 2023).

According to these participants’ narratives, despite rice consumption negatively affecting health, namely, as a cause of weight gain, the flavour, versatility in cooking, convenience, accessibility, and availability of various types of rice (market dynamics) contribute to the high consumption of rice. Respondents acknowledged that rice contains a high level of carbohydrates, which is why they linked it to weight gain, as has been observed in other studies on this macronutrient [70,71].

The familiarity with rice culinary practices may be explained because they were transmitted over generations, building the country’s gastronomy [57], and by the presence of a wide offer of rice varieties and types in the Portuguese market, allowing for a large culinary applicability. In the same way, versatility, positive sensory attribute evaluation, and positive perception of safety and speciality have contributed to increased rice consumption in Western societies [68,72].

The opposite was verified for brown rice, which is perceived as unpleasant, inconvenient, and a food not part of the traditional cuisine. These barriers to brown rice consumption were also found among consumers in Costa Rica [73]. Brown rice takes longer to prepare because the bran layer hinders water absorption [74], which will slow the meltdown of the crystalline structure of starch [75]. In addition, brown rice makes it difficult to achieve a good culinary result and has a higher unit price than polished rice, and the market is not so dynamic in terms of competing brands and promotions offered. As Selvam et al. [8] adverted, the low demand makes a product more expensive because of lower supply. Other studies have also identified the lack of convenience as a barrier to rice consumption, in which ready-to-eat rice formulations have been suggested to overcome the possible effects of this factor on rice intake [48,76,77]. This can be a valid solution, especially for *Carolino* and brown rice, the varieties considered more difficult to prepare. 

The main reason consumers buy brown rice and parboiled rice is that they are perceived as more nutritious, healthier, and more natural than milled rice. Brown or parboiled rice has a lower GI than its polished forms [30,78]. 

There was a positive perception of local rice’s quality, naturalness, and sustainability, which other cultures also consider when selecting local rice over imported rice [79]. This is in contrast with the continued decrease in the consumption of local rice, giving way to imported rice [3]. 

The sensory preference for rice differs among cultures [80,81,82,83], in which texture is considered the main sensory attribute in the consumer acceptability of cooked rice [83,84]. The grain’s length, width, and thickness were characteristics that participants weighed most when choosing raw rice, who believed that these attributes allow the prediction of the quality of cooked rice, a similar attitude of Asian consumers [85]. In the consumption stage, participants preferred rice with a soft and loose (non-stick rice) texture, like consumers in South Asia and other southern European countries, though unlike the rest of Europe, which prefers firmer rice [80,86]. Regarding appearance, participants preferred firm, thick grains (great swelling capacity), whole, closed (non-crack), and shiny grains, in agreement with the study by D’Hauteville et al. [87]. The panel expressed their preferences for each rice dish by describing the appearance of grains, flavour, and texture. Independent of the dish, they preferred whole kernels and grains with good extensibility (swelling of the cooked grain), which is considered an important cooking quality of rice by the Southern European consumer [86].

Baking, roasting, grilling*, estrugido*, braising, boiling, porridge, and stir-frying were the most prominent cooking methods reported, revealing familiarity with these cooking methods. *Estrugido* (Portuguese word) is a specific term for braising rice, which is frying garlic and onion in olive oil (may include other condiments) and adding colour without burning, where the other ingredients of the dish to be cooked are later added. Preferred cooking methods are usually associated with greater sensory benefits [67,88]. Thus, to cook rice, participants selected the best method for achieving the desired sensory properties, limiting the amount of water, and varying the rice-to-water ratio depending on the intended final dish, unlike consumers in northern Europe, who generally cook rice with large amounts of water and then discard the remainder of the water [86]. For example, French consumers prefer cooking in large amounts of water due to its ease and speed [67,88]. Cooking conditions such as temperature, time, cooking water, and pre-cooking steps such as soaking have been shown to affect the sensory properties of cooked rice [74,89,90]. Soaking improves texture, reduces cooking time, and improves safety [91]. In addition, pre-cooking steps, such as removing impurities from rice and soaking rice, are traditional practices in Japan, Korea, and other Asian countries [48,69]. The participants showed the habit of adding other ingredients when making rice; some of them did it to increase the nutritional value, and others added it to add more flavour. Masking the perceived unpleasant sensory characteristics of brown rice through the incorporation of ingredients and use in traditional dishes was one of the strategies indicated by Monge-Rojas et al. [73] to promote the consumption of brown rice among Costa Ricans. Similarly, in Korea, other cereals, vegetables, and/or pulses are traditionally added to rice to obtain more nutritious and healthy dishes [88,92]. This reinforces the similarities between Portuguese consumers and typical rice-consumer countries, as previously reported by Cabral et al. [57].

The preparation and eating stages also influence the initial decision to buy rice (acquisition stage): consumers choose according to the desired culinary result, available time, cooking skills, and market opportunities (price and rice types). These factors confirm that Portuguese consumers are mainly motivated by the utilitarian and experiential dimensions of rice [57]; in the sense that the value of rice is determined by how well it performs, namely, considering its use as a side dish or main course, as a source of nutritional properties, or as a means of preparing a conventional meal, as well as by the fact that they appreciate the sensory properties of rice, as it offers pleasure and evokes feelings of pleasure [93].

### 5.2. Determinants of Choice of Low-GI Rice

For free word association with the “low glycaemic index foods” stimulus and the words related to health and benefits of low-GI foods, “Sugar” was the sixth most cited word, generating a double interpretation. On the one hand, this could be a sign that some participants were completely unaware of the concept of GI; on the other hand, it could be a direct association between sugar and what GI is, and not necessarily a mention of sugar as a low-GI product. Therefore, in-depth interviews are important for clarifying this issue.

In the interviews, we verified that to define GI, several participants answered “… it has to do with sugar.” Although some participants were unaware of this concept, the erroneous association was related to the GI of fat and food colour. Those who mentioned sugar believed that there was a dependence between GI and sugar (not to classify the GI of sugar), since the glycaemic index depends on the amount and nature of the sugar contained in food [20].

Participants associated food items such as “Fruits and vegetables”, “Whole grains”, “Dairy products”, “Fish and white meat”, and “Pulses and seeds” to the stimulus, which are foods consensually perceived as healthy and recommended in the main healthy eating guides [94]. Whole grains have been spontaneously connected to health, even when studying terms or phrasal stimuli without product exposure [95]; therefore, these categories reinforce a positive perception of the health benefits associated with the “Low GI food”. The category “Fish and white meat” was formed predominantly of fish (only 6 out of 25 mentions were for white meat), which is justified by the familiarity with this food, since Portugal is one of the largest consumers of fish in Europe [96].

Older participants expressed more “Positive attitudes and emotions” when hearing about “foods with low GI” than younger participants. In this age group, we believe that even if they did not have full knowledge of the concept, there was some idea of the benefits that might have been gained from medical contact (it can be part of medical advice in routine appointments). This finding corroborates previous studies that have reported that ageing is associated with greater awareness of the relationship between health, diet, and disease [97,98]. Participants with a higher education showed greater knowledge of the concept of low GI than those without a higher education. This result is in line with those already mentioned by several authors that demonstrated the influence of the level of education on knowledge about healthy eating [99,100]. These findings reinforce that foods with special claims should be designed for specific groups rather than targeted at the general market.

Some participants understood the low-GI concept and its impact on health, while others were misinformed with huge misconceptions. Lack of information/knowledge was identified as a barrier to choosing rice products with a low GI. These results suggest greater reflection on the role of information in the promotion of low-GI rice, both to inform about the product and to raise awareness about the concept. Correcting inaccurate beliefs regarding nutrition can improve the nutritional quality of choices [101]. For example, Arslain et al. [102] show that even a short message about the benefits of fibre significantly increases consumption of dietary fibre; Gustafson et al. [103] showed that more specific prompts about healthy foods at the point of decision can motivate healthier choices; and Jo and Lusk [101] found that information about health benefits makes consumers more willing to sacrifice their tastes for healthy foods. Urala and Lähteenmäki [104] found that some health claims can be so strong that consumers are ready to compromise on taste. However, this depends on the consumption values and degree of interest in that claim. We believe that consumers affected by diabetes or other diseases that can be controlled through food intake would be more willing to make these sacrifices, since it is recognised that the diagnosis of health problems related to food intake is one of the exceptions to the domain of hedonic reasons for one’s food choices [105]. Therefore, including information on the label, such as short and simple explanations about the impact of a low-GI diet, prompts at the point of sale, and health goal priming can be a potential tool for promoting rice/low-GI products.

Participants showed an interest in low-GI rice-based healthy products that offer convenient solutions without compromising taste and price, showing concerns about more immediate aspects of food consumption decisions, such as taste and price [106]. As participants idealised these products as convenient and healthy, they perceived them as expensive and tasteless. Increasing convenience (time savings, ease of preparation, energy savings, etc.) adds value to the final product, which translates to a higher price [107], and healthy foods are perceived as more expensive [102]. In some cultures, there is an intuition that healthy foods are not tasty [108,109]. Consumers have even reported this belief with a low food pleasure orientation [110].

The naturalness emphasised by participants may be related to the desire for convenience foods to be the closest to homemade foods, as verified by Peura-Kapanen, Jallinoja [111]. The lack of naturalness has been reported as a barrier to consuming conventional food substitutes [112]. For many years, convenient products were perceived as unhealthy [111]. However, changes have reinforced the use of convenience as a tool to support healthy eating habits [113].

Studies continue to support using low-glycaemic index diets as a treatment for diabetes [23]. However, the potential applications of these diets extend beyond diabetes management, and they may also be effective in preventing and managing other chronic diseases, including cardiovascular disease, some types of cancer, and obesity [114]. Taking advantage of the country’s culinary habits, using ingredients that can potentially lower the glycaemic response to rice meals can be encouraged. The method of adding ingredients such as vegetables, beans, and ancient grains has been used to obtain rice dishes with a lower GI [40,42,43,44,45]. Moreover, Chang et al. [115] found that adding vegetables when cooking rice increased satiety and decreased daily energy density, owing to the reduced amount of rice consumed in the meal. This can be a good strategy for weight control and the maintenance of diabetes.

Rice has proven to be a raw material with great potential, owing to its profitability, ease of transportation for strengthening nutrients, versatility, and ability to be integrated into conventional formulations or specific products for consumers with special diets, such as celiacs. Therefore, the food industry plays an important role in developing and promoting healthier ways of consuming rice, whether through product formulations resembling conventional products or using traditional ingredients with which consumers are familiar.

Researchers and industries have investigated the processes of the nutritional optimisation and reduction of the GI of rice, such as germination, which gives rise to the so-called GABA rice—rice with a natural content of gamma-aminobutyric acid, a neurotransmitter with calming effects on the brain, fermentation to obtain rice-based probiotics such as Amazake, and ready-to-eat rice meals mixed with beneficial ingredients such as ancient grains, pulses, and vegetables [54,116,117,118,119].

## 6. Conclusions

Unlike most European countries, rice is an almost daily staple for Portuguese consumers, and rice remains an important part of Portuguese cuisine. The factors considered in the choice of rice vary throughout the rice supply and use method. Consumers tend to try new varieties, listing sensory aspects, price, and convenience as preponderant factors. The participants’ narratives suggest that three main factors may explain why this consumption frequency is so high in comparison to other European countries: (i) rice was considered convenient for different culinary situations (cooking stage), (ii) the taste was very well accepted (eating stage), and (iii) the high cultural/traditional identity associated with rice consumption in Portugal, which is also observed in the market dynamics (acquisition stage).

Low-GI rice foods/products were considered interesting and healthy due to their perceived nutritional and general health benefits. On the other hand, consumers were concerned about their sensory dimensions. Therefore, sensory analysis using consumer panels is essential for developing low-GI rice products. As traditional habits and naturalness are drivers of the willingness to choose low-GI rice foods, food manufacturers must take heed to offer healthier rice products that resemble conventional rice dishes. To achieve this, it is essential to employ processing technologies perceived as natural, and to incorporate sustainable natural ingredients commonly used in everyday cooking.

Moreover, the findings suggest that educational actions should be considered to change negative perceptions of brown rice and low-GI foods. Examples of such actions could be the dissemination of diversified recipes that include brown rice and a strategy for preparing white rice with ingredients that help reduce GI, considering the gastronomic value of the ingredients for the Portuguese population. GI labelling can be a valuable tool for the promotion of such products. For a successful launch of a rice product with a low GI, it is necessary to invest in disseminating its benefits and to bring it as close as possible to a familiar product for the consumer.

The insights from this study can be used in future research with a more quantitative approach to evaluate the factors underlying the choice of rice and low-GI rice products, the most attractive sensory and non-sensory attributes, and the segmentation of consumers willing to choose this type of product. Regarding ways to promote rice consumption in its healthiest form, it is important to understand the impact of information about GI and the awareness of healthier rice alternatives in shaping rice choices.

## Figures and Tables

**Table 1:** Overall self-reported rice consumption and according to rice type.

Rice Consumption	Rice *	*Agulha*	*Carolino*	Basmati	Parboiled	Brown	Jasmine	Risotto
<One time/week	1.2%	20.3%	36.8%	62.9%	74.6%	84.4%	87.9%	93.4%
One to four times/week	59.0%	64.5%	54.3%	36.6%	20.7%	14.1%	10.9%	6.7%
Five or more times/week	39.8%	15.3%	9.0%	3.6%	4.7%	1.6%	1.2%	0.0%
Average (±SE) ***	4.4 ± 0.16	2.6 ± 0.14	1.9 ± 0.14	1.1 ± 0.10	0.8 ± 0.09	0.5 ± 0.07	0.4 ± 0.05	0.3 ± 0.02

*** Average (± SE) weekly rice consumption in general and by rice type. * Overall, all types included. Sample size = 256.

**Table 2:** Frequency of participants (%) who self-reported rice consumption according to the place of consumption.

Rice Consumption	Home (*n* = 256)	Restaurant (*n* = 186)	Canteen (*n* = 62)	Other * (*n* = 57)
<One time/week	0.8%	57.0%	24.2%	35.1%
One to four times/week	64.9%	37.1%	61.2%	47.4%
Five or more times/week	34.3%	5.9%	14.5%	17.6%
Average weekly consumption (±SE)	4.1 ± 0.14	1.0 ± 0.13	0.9 ± 0.35	0.9 ± 0.44

* Relatives and friends’ homes. Sample size = 256.

**Table 3:** Frequency of participants (%) who self-reported purchasing different commercial formats of rice products.

Rice Purchase	Raw	Takeaway	Dehydrated	Pre-Cooked	Frozen	Refrigerated
<One time/week	42.3%	77.3%	93.4%	93.7%	98.0%	96.9%
One to four times/week	40.6%	21.5%	6.6%	6.3%	1.6%	1.9%
Five or more times/week	17.1%	1.2%	0.0%	0.0%	0.4%	1.2%

Sample size = 256.

**Table 4:** Frequency of words (%) and participants (%) according to the dimension and category of the “Low GI foods” stimulus.

Dimension	Category	Examples of Elicited Words	Word (%)	Respondent (%)
Fruits and vegetables	Vegetables	broccoli, carrots, greens, lettuce, spinach, sweet potatoes, vegetables, and watercress.	13.9	32.8
	Fruits	apple, fruit, lemon, pear, watermelon.	7.4	21.1
Nutrition	Nutritional aspect	balanced diet, dietary, carbohydrates, correct diet, diet, eat tiny amounts, energy, healthier eating, nutrition, restriction, routine, water	10.7	26.6
	Sugar reduction	eat little sugar, little sugar, low in sugar, low carbohydrates, low consumption of sweets, no sugar, sugar-free	3.9	10.5
	Low GI disconnects	healthy for the intestines, light, low-fat, low fibre, moderate salt, no salt, vitaminic, without fat, with iron	3.3	8.2
Health	Health	health, healthy	8.6	23.0
	Physiological	age, blood glucose, diabetes, slow absorption, hunger	5.9	14.5
Whole grains	-	brown rice, oat, whole grains	7.7	21.5
Positive attitudes and emotions	-	advisable, balance, beneficial, comfort, decisive, determining, desire, good mood, happy, happiness, ideal, living better, necessary, satisfaction, quality, quality of life, positive, wellbeing, vitality	6.9	15.2
Sugar	-	fructose, glucose, sugar	4.0	9.8
Dairy products	-	cheese, dairy products, milk, yoghurt	3.6	10.5
Bread and pasta	-	bread, pasta	3.4	9.4
Fish and white meat	-	chicken breast, fish, white meat	3.3	8.2
Pulses and seeds	-	almonds, beans, chickpeas, lentils, peanuts, pulses, seeds, soybeans, walnuts	3.5	8.6
Sensory	-	bitter, flavour, sour, taste, tasteless, white	2.9	7.4

Simple size = 256.

**Table 5:** Respondents’ absolute frequency in the free word association task using the “Low GI foods” stimulus, according to the elicited categories and gender, age group and educational level.

Dimension	Gender		Age Group (Years)			Educational Level	
Category	Male	Female	[18; 34]	[35; 54]	55+	No Higher Education	Higher Education
**Fruits and vegetables**	45 (−)	116 (+)	62	81	18 (−)	97	64
Vegetables	30	75	41	52	12	60	45
Fruits	15	41	21	29	6	37	19
**Nutrition**	58 (+)	77 (−)	55	57	23	87	48
Nutritional aspect	38 (+)	44 (−)	37 (+)	33	12	50	32
Sugar reduction	13	16	14	11	4	17	12
Low GI disconnects	7	17	4 (−)	13	7	20 (+)	4 (−)
**Health**	45	66	34	55	22	76	35
Health	27	39	21	30	15	46	20
Physiological	18	27	13	25	7	30	15
**Whole grains**	16	43	25	27	7	30 (−)	29 (+)
**Positive attitudes and feelings**	22	30	7 (−)	28	17 (+)	33	19
**Sugar**	14	16	7	17	6	17	13
**Dairy products**	14	14	14	12	2	18	10
**Pulses and seeds**	13	13	9	12	5	18	8
**Bread and pasta**	7	19	8	13	5	5 (+)	**21 (−)**
**Fish and white meat**	8	17	6	14	5	17	8
**Sensory**	7	15	13 (+)	7	2 (−)	10	12

(+) or (−) indicate that the observed value is significantly higher or lower than the expected value (χ^2^ test per cell with a significance level of 0.05). Sample size = 256.

## Data Availability

Data is contained within the article or Supplementary Materials.

## Supplementary Materials

The following supporting information can be downloaded at: https://www.mdpi.com/article/10.3390/foods13020301/s1, File S1: In-depth interview guide on determinants of the consumption of rice and products with a low glycaemic index; File S2: self-reported electronic questionnaire.

## Appendix A

**Table A1:** Characterization of the participants in the questionnaire.

Characteristic		Absolute Frequency	Relative Frequency
Gender			
	Female	164	64%
	Male	92	36%
Age group (mean ± SD: 40 ± 12.8 year)			
	[18–34]	90	35%
	[35–54]	124	49%
	≥55	42	16%
Educational level			
	no higher education	164	64%
	higher education	92	36%
Household size			
	1 and 2	77	30%
	3 and 4	158	62%
	5 or more	21	8%
Monthly household income per capita			
	EUR ≤ 250.00	65	25.4%
	EUR [250.01–400.00]	66	25.8%
	EUR [400.01–550.00]	62	24.2%
	EUR > 550.00	63	24.6%

Sample size = 256.

**Table A2:** Characterization of the participants in the interviews.

Consumption Group (Average Age ± SD)	Participant (P#)	Gender	Age Group	Education Level
G1—Daily rice consumers (minimum consumption once a day)	P1	Female	[35; 54]	no higher education
	P2	Female	[18; 34]	no higher education
	P3	Male	[35; 54]	no higher education
	P4	Male	≥55	higher education
	P5	Female	[35; 54]	higher education
	P6	Female	[35; 54]	no higher education
	P7	Male	[18; 34]	no higher education
	P8	Male	[18; 34]	no higher education
G2—Light rice consumers (consumption of rice up to three times/week)	P9	Female	[35; 54]	higher education
	P10	Female	[35; 54]	no higher education
	P11	Male	≥55	no higher education
	P12	Female	[35; 54]	no higher education
	P13	Male	[35; 54]	no higher education
	P14	Female	≥55	no higher education
	P15	Female	[18; 34]	higher education
	P16	Male	[18; 34]	no higher education
G3—Brown rice consumers (consumption of at least three times/week of brown rice)	P17	Female	≥55	no higher education
	P18	Male	≥55	no higher education
	P19	Male	[35; 54]	no higher education
	P20	Female	[35; 54]	higher education
	P21	Female	[18; 34]	higher education
	P22	Female	≥55	no higher education
	P23	Male	[18; 34]	no higher education
	P24	Male	[18; 34]	no higher education

Sample size = 24.

**Table A3:** New rice-based products as cited by participants during the in-depth interviews.

Type of Rice Products (No. of Participants)	Rice Products (No. of Participants Who Cited the Products)	Example of Quotations
Products recently tasted by participants (*n* = 18)	Puffed rice cake and crackers (*n* = 15)	‘*I also buy rice cake. They’re white, I call them Styrofoam, it doesn’t even remind me that it’s rice. I quite like it*’, P9
		*‘Sometimes I buy [crackers] to take with me when I’m going to be away from home for a long time, so I take it with me’*, P6
		*‘It’s great, it’s great! They are delicious [rice cake]. It really has cocoa; the chocolate is made from cocoa’*, P14
		*‘…chocolate rice cake, rice crackers. These are the ones I’ve tried’*, P15
	Rice drinks (*n* = 8)	*‘One thing I drink every day, rice drinks because at home I try to cut out lactose’*, P20
		*‘…rice milk. I happened to have a drink last week’*, P7
		*‘Rice drink, we’ve already tasted it’*, P13
	Ready-to-eat (*n* = 4)	*‘I liked vegetable risotto’*, P3
		*‘I really like new products (…) we tried noodles, noodles with egg. We like it because I just add hot water’*, P19
		*‘What I bought new was rice that is prepared in the microwave (…) cod rice’*, P22
		‘*I have already used the ready-to-eat, especially the plain rice. It’s just that I have a problem with [to make] plain rice: it never comes out well. I use it when I want it to look really good. It goes in the microwave, and it’s done, and I don’t have to worry’*, P15
	Rice flour and noodles (*n* = 4)	*‘…rice noodles, for example, is something that I like’,* P4
		*‘I have used rice flour’*, P5
		*‘I use rice flour a lot, I like it a lot’*, P17
Product concept proposed by participants (*n* = 15)	Snack and breakfast cereal (*n* = 7)	*‘Cereal is what I always eat at home from morning to afternoon and before bed too. And since it has a lot of sugar, if there was one with a lower glycaemic index, maybe I would opt for this product instead of the conventional one’*, P8
		*‘Crackers always go well. And if it has a low glycaemic index all the better. At least I eat crackers and I was quenched for longer’,* P12
		*‘For example, cereal bar. Of those bars that you sometimes buy with mixed fruit. Some different bars with [puffed] rice’*, P20
		‘*A snack would be interesting. Like a bar. People are looking for a lot of bars and the bars are loaded with sugar. It is very difficult to find a healthy one’*, P21
		*‘Rice-based breakfast cereals, kind of oat’*, P24
	Ready-to-eat meal/side dish (*n* = 6)	*‘…for me a faster meal because of gaining time (…) Rice noodle, I would eat it often. Pasta, pizza’*, P3
		*‘An easy-to-prepare rice’*, P9.
		*‘Ready-to-eat rice, not to use regularly, but to save time when necessary’*, P16
		*‘I really like rice, I like a lot of hearty food, (…) if it’s a good seafood rice, I don’t know, a risotto, a good risotto’*, P18
	Desserts and dairy substitutes (*n* = 5)	‘*Yogurt containing rice would be interesting. I don’t know, it’s a suggestion (…) A milk-like drink that had some components like fruit. That it was more consistent than the rice drink. Like yogurt. I think something like that was needed [for eating] there in the middle of the afternoon’*, P1
		*‘Only if it’s in the form of desserts, (…) as we want to get away from the traditional dessert, maybe, having a low glycaemic index we say: ok, this is good’*, P5
		‘*A smaller yogurt-sized product I think would be interesting*’, P6
		*‘…rice drink. I shop at stores that sell healthy products. If it is a rice drink with a lower glycaemic index, I would opt for the healthier one’,* P8

Sample size =24.
