# Supplementary material for: Exploring Rice Consumption Habits and Determinants of Choice, Aiming for the Development and Promotion of Rice Products with a Low Glycaemic Index
Source: Foods. 2024 Jan 17;13(2):301. doi: 10.3390/foods13020301 (PMC10814881; doi:10.3390/foods13020301)
Supplement: Supplementary file 1 [file foods-13-00301-s001.zip › foods-2768618-supplementary.pdf]

Supplementary information to:

Exploring rice consumption habits and determinants of choice, aiming for the development and promotion of rice products with low glycaemic index

Diva Cabral [1,2], Ana P. Moura [1,3], Susana C. Fonseca [1,2], Jorge C. Oliveira [4] and Luís M. Cunha [1,3*]

File S1: In-depth interview guide on determinants of the consumption of rice and products with alow glycaemic index

Topic: Evaluation of rice consumption habits, determinants, and expectations of low glycaemic index (GI) foods.

Objectives:

- To identify the key drivers and barriers affecting rice consumption.
- To assess the interviewees' perspectives on new rice products in the market and understand the factors that influence or limit the acceptance of these products.
- To evaluate the interviewees' perceptions of rice and rice-based products with a low glycaemic index. Understand expectations regarding rice or rice-based products with a low glycaemic index.

Profile of interviewees: 3 groups of 8 people who responded to the first survey.

Group 1 - Dairy consumption (who consume rice at least once a day)

Group 2 - Light rice consumers (who consume rice less than three meals per week)

Group 3 - Brown rice consumers (who consume at least three brown rice meals per week)

Location of interviews: Sense Test, Lda. Rua Zeferino Costa nº 341, Vila Nova de Gaia

Resources: Experienced moderator, guide with a list of questions to ask, two reporters to collect data throughout the interview: video/audio recorder, pen, and notepad.

Comfortable, friendly atmosphere.

Introduction (welcome, thanks, topic overview, and interview ground rules)

My name is (…), and I will conduct this interview on the determinants of purchasing rice and rice-based foods (with a low glycaemic index). First, thank you for your participation. In this interview, we will try to capture the main reasons for consuming rice. It is also intended to understand the interviewee's position in relation to rice products with a low glycaemic index. Your opinions and comments were valuable for achieving our goal. I remind you that there are no right or wrong answers; these are opinions that will only be used anonymously for research. All opinions were welcome.

With your permission, the interview will be recorded. First, I would like you to introduce yourself, and say your name, age, and occupation."

I propose that before we start our conversation, you perform the following exercise: identify in as much detail as possible what you ate in the last week, whether for lunch or dinner. Please fill in the following grid.

File S2: Question

A- Rice choice criteria and consumption habits

1) Does everyone in your house consume rice? (*if someone doesn't consume it, understand why and their profile*).
2) What type of rice do you usually buy? (*Understand the reasons for choosing each type of rice and understand the specific sensory characteristics that*

motivate each choice. Understand if there are a variety of choices for special dates or days).

3) How often do you consume brown rice? Why? (*If you consume it, understand the reasons, the characteristics you like, and how you cook it. If you do not consume it, find out why, and if there is any possibility of consuming it*).
4) When you buy rice, what factors do you consider when purchasing? (*Develop this issue as much as possible, considering the main determinants of consumption*).

   (a) What do you normally do when you go shopping and find rice on sale? (*Understand the extent to which promotions make people choose these products instead of their usual or preferred products. In addition, we understand whether promotions increase the quantity of the product in the basket. Understand whether there is a preference for the place of purchase, understand what they value, and do not value in a package: material, shape, weight, label, whether the origin and brand are important*).

B- Rice sensory criteria valorisation

1) What characteristics do you most appreciate for rice? (*Understand valued dimensions*).
2) How do you like white rice? How do you want its appearance?
3) How do you like a rice dish that contains more ingredients in its recipe (in addition to the ingredients used to cook plain rice)?

C- knowledge about foods with low GI and drivers of choice of rice with low GI

1) Do you take any specific care of your diet? (Note: if the participant has already mentioned this in the previous section, move on to the following question)
2) How does the nutritional composition of rice and rice-based products influence your choice? (*Understand what the concerns are in terms of the nutritional content of rice*).
3) Have you heard about the glycaemic index of foods? You know what it is?

The glycaemic index (FAO/WHO, 1998) is defined as the increase in blood glucose after eating a certain food. (*Provide examples for better understanding*: Examples of high GI foods include wheat white bread, potatoes, and sugary desserts; in contrast, foods characterized by a smaller increase and decrease in blood glucose, fresh fruits, and vegetables such as apple, pear, and beans were exemplified).

4) What do you know/think about the GI of rice?

D- Expectations regarding new rice products

1) When new rice-based food products are introduced to the market, do you usually buy them?
2) What would lead you to choose a new product over a traditional product?
3) Would you be willing to consume rice products with a low glycaemic index? What would make you buy a rice product with a low glycaemic index?
4) What would a rice product with a low glycaemic index (healthier) must interest you?
5) How do you think of or design this product? (Ideas for new products).
